# A New Method to Test Molluscicides against the Philippine Schistosomiasis Snail Vectors

**DOI:** 10.1155/2020/3827125

**Published:** 2020-01-29

**Authors:** Darwin C. Gomez, Neil Anacta

**Affiliations:** ^1^Biochemistry and Schistosomiasis Research Laboratory, Natural Sciences Department, College of Arts and Sciences, Eastern Visayas State University, Tacloban City 6500, Philippines; ^2^Institute of Chemistry, College of Science, University of the Philippines, Diliman, Quezon City 1101, Philippines

## Abstract

To expedite the discovery of novel molluscicides in the laboratory, this study aimed to evaluate the performance of a new molluscicidal assay. This assay is based on *Oncomelania hupensis quadrasi* snails and is called miniaturized plate test or *mpt*. To perform this assay, a 12-well plate, 3 snails per well, and 24-h exposure period were used. The performance of *mpt* was evaluated using niclosamide and *Ardisia* plant extract (tagpo extract) as test substances while WHO's guidelines for a conventional plate test (*cpt*) served as standard. One *cpt* and four *mpt* independent trials were performed for niclosamide and tagpo extract. Probit analysis of dose–response data was run in R to generate lethal concentrations (LC_50_ and LC_90_), while lethal ratio test was performed to detect significant difference between paired LC_50_s (or LC_90_s). Using niclosamide, the calculated LC_50_ values were 0.104, 0.127, 0.136, 0.139, and 0.140 g/m^2^ for *cpt*, *mpt* 1, *mpt* 2, *mpt* 3, and *mpt* 4, respectively, while the LC_90_ values were 0.266, 0.268, 0.244, 0.251, and 0.261 g/m^2^, using the same sequence, respectively. For tagpo extract, the LC_50_ values were 1.467, 1.547, 1.659, 1.797, and 1.659 g/m^2^, for *cpt*, *mpt* 1, *mpt* 2, *mpt* 3, and *mpt* 4, respectively, and the LC_90_s were 2.188, 2.195, 2.501, 2.358, and 2.501 g/m^2^, respectively. The lethal ratio test revealed that a significant difference exists between the LC_50_s of *cpt* and *mpt* 1 when using niclosamide with a lethal ratio and confidence limits of 0.820 (0.663, 0.977, *p* < 0.05) and another significant difference between LC_50_s of *mpt* 1 and *mpt* 3 using tagpo extract with computed lethal ratio and confidence limits of 0.861 (0.782, 0.939, *p* < 0.05). Taken together, the results point out that *mpt* generates accurate and reproducible lethal concentration values. Hence, *mpt* may be used as an alternative method to screen molluscicides that are active against schistosome snail vectors.

## 1. Introduction

Population control of disease vectors is an important strategy to manage schistosomiasis, a parasitic disease caused by schistosome worms and transmitted by freshwater snails [1, 2]. According to a recent modeling study, mass chemotherapy combined with snail population control is an economical strategy to eradicate schistosomiasis [3]. This mathematical model is supported by a recently published study in China. A longitudinal analysis was performed using 37 years worth of research data on schistosomiasis in Sanlian and Guifan, China [4]. In that study, a combined mass administration of praziquantel and snail control strategies resulted in significant reduction of human prevalence of schistosomiasis [4]. In China, snail control strategies include molluscicide application, environmental management, and land conversion [5]. These strategies may be difficult to implement if snail sites vary in size and terrain or when molluscicide being used is toxic [5, 6]. For example, the use of synthetic molluscicides such as niclosamide poses environmental and health risks which necessitates the development of green alternatives [6]. The broad toxicity of niclosamide to many nontarget organisms such as fish and amphibians is the major concern against its use in mass application [5]. Thus, there are new attempts to develop novel molluscicides by optimizing the activity of known substances or tapping diverse compounds from natural sources [7–10].

A fast *in vitro* molluscicidal assay may accelerate the development of novel molluscicides. There is a growing interest in phytochemicals with potent molluscicidal activity after the successful use of *Phytolacca dodecandra* in the snail population control of schistosome vectors in Egypt [7, 8, 11]. Synthesis of new compounds and structure–activity relationship with known molluscicides are also gaining popularity [9, 10, 12, 13]. Both research strategies will require an assay which uses a small amount of test substance while maintaining accuracy and reproducibility. Recently, it has been shown that 2 mL of test solution in a 24-well plate can be used to screen molluscicides active against *Biomphalaria* species [14]. This new method was compared to the conventional protocol, a method where more than 50 mL of test solution is used for each technical replicate. Both methods were shown to generate statistically similar results [14]. The authors showed that the reduced volume of test solution needed in their new assay, in turn, reduces the amount of test substance required to generate dose–response data, making it suitable for natural products research where purified samples are always limited in amount [14].

Apart from the immersion method, molluscicides may be tested using the plate test. It is used for amphibious snails such as *Oncomelania hupensis quadrasi*. According to the World Health Organization's published guidelines for testing molluscicides in the laboratory, a plate test uses a 150-mm Petridish, an ordinary filter paper as flooring, ten (10) snails per plate, and a four-day exposure period [15]. These guidelines are adapted in various ways. Its modifications may include smaller Petridish sizes, shorter exposure period, and incorporation of a revival stage where snails are allowed to recover in favorable conditions. Even if the plate test method is simple, it suffers from a large amount of test substance required in testing and extended evaluation time. This means that the plate test may be impractical when used to test bioassay-guided fractions of complex mixtures and may be difficult to reproduce as an early onset of snail death may result in microbial growth and contamination of the plate. Plate contamination may be addressed by separating out dead snails or transferring those that are alive in a new plate. To add, in WHO's testing guidelines, statistical treatment of dose–response data is not explicitly described.

Robust and appropriate statistical treatment of dose–response data is important in assay development. Statistical treatment of data is used to model the distribution of responses sought in the assay (i.e., snail mortality rates) versus a range of concentrations of test substance. For example, Probit analysis which is used in lethal concentration calculation requires that experimentally derived dose–response data are fitted to a lognormal distribution [16]. The fitting of dose–response data depends on the model chosen and the experimental design [17]. According to Robertson et al. [18], a bioassay must use at least five concentrations and about 250 test organisms. The quality of dose–response data is assessed using goodness-of-fit tests and significance testing is performed using lethal ratio test [18, 19]. In this study, robust statistical treatment of data derived from a proposed novel molluscicidal assay was performed to support its accuracy and reproducibility in calculating lethal concentrations.

This study aimed to develop a miniaturized version of WHO's plate test called the miniaturized plate test (*mpt*) based on *Oncomelania hupensis quadrasi* snails. To test the hypothesis that no significant difference exists between the lethal concentrations calculated using *mpt* and the conventional plate test (*cpt*), the reference molluscicide niclosamide and a novel plant extract were used as test substances. Then, to evaluate the reproducibility of *mpt*, multiple trials were performed. Probit analysis was used in lethal concentration calculation, while lethal ratio test was employed in hypothesis testing [16, 18].

Moreover, this study used the crude plant extract from an *Ardisia* species which is currently being studied by the authors for its potential molluscicidal activity against *Oncomelania hupensis quadrasi* snails (unpublished data). This plant is a native species in the Philippines and commonly known as “tagpo”. Many *Ardisia* species are well studied for their health-promoting activity [20]. The purpose of including tagpo extract as a test substance is to show that *mpt* can be used to test crude plant extracts.

## 2. Materials and Methods

### 2.1. Preparation of Niclosamide

A powder formulation of niclosamide (50% by weight) was purchased from a local dealer (NiclosM®, 50WP, Leads Agricultural Products Corp.). Seven niclosamide concentrations were used in the assay ranging from 0.0617 g/m^2^ to 0.2330 g/m^2^ of the active ingredient using water as solvent. To prepare the stock solution of niclosamide, 0.3530 g of 50WP niclosamide was weighed in an analytical balance (Shimadzu, Japan) and dissolved in 1 liter of distilled water using a volumetric flask. The solution was spun at 1900 × *g* for 4 minutes in a benchtop centrifuge (Labnet, USA). The centrifugate was stored in 50-mL centrifuge tube and stored at 4–8°C until used.

### 2.2. Preparation of Plant Extract

Tagpo fruit samples were collected in Sta. Fe, Leyte, Philippines (11° 11′ 52″ N, 124° 56′ 29″ E, 43 m altitude) during the months of November and December 2018 and were identified as an *Ardisia* species at the Jose Vera Memorial Herbarium, University of the Philippines, Quezon City. The binomial name of the species was not determined due to the complexity of the genus. Green–colored tagpo fruits were brought to the lab and washed in running tap water to remove dirt. The fruits were cut into half and dried at 60°C for 24 h. Dried fruit samples were then pulverized using a blender. The pulverized fruit samples were sieved and stored in 50-mL centrifuge tubes at −21°C until used. Crude tagpo extract was prepared by soaking 1.000 g of pulverized samples in 100.0 mL of distilled water. The solid–liquid mixture was stirred continuously for 1 h and was spun at 1900 × *g* for 4 minutes using a benchtop centrifuge (Labnet, USA) at room temperature. The centrifugate was stored in 50-mL centrifuge tubes and used in downstream molluscicidal bioassays. The concentration of tagpo extract was expressed in grams of soluble solids per plate area (g/m^2^) and determined using gravimetric analysis of the centrifugate following the method of the American Public Health Association [21]. For tagpo extract, five concentrations (1.224 to 2.396 g/m^2^) were used.

### 2.3. Acclimatization of Snails

Snails (*O. h. quadrasi*) were collected in Alang-alang, Leyte (11° 11′ 19″ N, 124° 54′ 23″ E, 49 m altitude) and acclimatized to laboratory conditions in a glass aquarium lined with filter paper on inner walls. Dechlorinated water was used for acclimatization. The water inside the aquarium was maintained at a 5 mm water depth and the water was changed every 24 h. The filter paper served as food for the snails. The snails were acclimatized under 12 h : 12 h light : dark cycles for 48 h.

### 2.4. Conventional Plate Test (cpt)

Molluscicidal assay was performed using 85 mm Petri plates fitted with circular filter paper on the plate surface. Appropriate volume of niclosamide or tagpo extract was distributed evenly on the filter paper. Then, the Petri plates were dried in an oven at 60°C for 6 h and cooled to room temperature. Ten snails were placed in the center of the plate and the test substance was reconstituted by distributing evenly with 2 mL of distilled water. Each test concentration was paired to one negative control. There were as many plates for the negative control as the number of concentrations used, meaning seven plates for niclosamide and five for tagpo extract. The plates were checked after 1 h, and snails climbing on plate walls were returned back in the center of the plate by means of tweezers. After 24 h, the snails were assessed for mortality.

### 2.5. Miniaturized Plate Test (mpt)

The assay was performed using 12-well plates made from polyethylene material. The wells were fitted with 21.5 mm circular discs of filter paper. Nine out of 12 wells were added with appropriate volumes of niclosamide and tagpo extract using a micropipetor. The well plates containing the test substances were dried in an oven at 60°C for 6 h and then allowed to cool to room temperature. Three snails were added per well, a total of 36 snails were used per plate. Nine wells were used for a test substance and the remaining three wells were used as negative controls. Then, 130 microliters of distilled water was added to each well to reconstitute the test substances or to make the negative control. Similar to *cpt*, well plates were checked after 1 h and snails adhering on the wall were returned to the center of the well using tweezers. Snail mortality was assessed after 24 h.

### 2.6. Assessment of Snail Mortality

After 24 h of exposure to tests substances, snails in Petri plates or 12-well plates were assessed for mortality using motility criterion. In this study, snail recovery step was skipped since we were able to assess motility of snail tissue under the stereoscope. Aside from motility criterion, the degree of decomposition and its associated foul smell were also used for mortality assessment. To determine the count of dead snails, the snails were placed separately on a clean glass slide followed by a drop of water to each snail. The snails were crushed gently with another glass slide to expose snail tissues. The exposed tissues were prodded with a needle and lack of response after prodding was used as the mortality criterion. As validation, we always observed noticeable reaction among snails in negative controls upon stimulation with the needle tip, that is, after crushing between two glass slides.

### 2.7. Statistical Analysis

Probit analysis or the Finney method was used to determine the median and maximal lethal concentrations (LC_50_ and LC_90_, respectively), Chi-squared values, lethal ratios, and their corresponding 95% confidence limits using the *drc* package in R [16, 22, 23]. A significant difference was detected in the lethal ratio between LC_50_s (or LC_90_s) when the confidence limits of the ratio *did not* include 1.000 [18].

## 3. Results

In this study, a miniaturized version of WHO's conventional plate test (*cpt*) was developed. This new method is termed as the miniaturized plate test or *mpt*, which was performed with a 12-well plate (21.5 mm diameter), 3 snails per well, and 24 h exposure period to generate dose–response data. One trial of *cpt* and four trials of *mpt* were performed using seven test concentrations of niclosamide and five of tagpo extract. The researchers used WHO's conventional plate test as standard.

From the initial concentration of niclosamide and tagpo extract and the volumes used in the assay, the amount of substance used was calculated. The decrease in plate area in *mpt* resulted in a smaller amount of substance required to perform the assay. The amount of niclosamide used in *mpt* (0.0035 g) was five times lower than in *cpt* (0.0171 g) (Table 1). Similarly, for tagpo extract, only 0.0303 g was used for *mpt* and 0.1459 g for *cpt*. Moreover, a smaller number of snails was used in *mpt* than in *cpt*: 252 snails for *mpt* versus 280 for *cpt* with niclosamide and 180 snails for *mpt* versus 200 snails for *cpt* with tagpo extract (Table 1).

All ten sets of dose–response data are presented in Table 2. The molluscicidal activity of niclosamide and tagpo extract was dose-dependent which means that more snails were counted as dead at higher concentrations. No snail mortality was observed in negative controls for niclosamide and tagpo extracts, which implied that the snails were competent and the mortality recorded with test substances was due to their molluscicidal action. These results also supported that our method of snail mortality assessment by snail crushing generated accurate and reproducible dose–response data. Notably, we have observed that more snails climbed out of the petriplate or well plates in niclosamide especially at lower test concentrations (i.e., 0.1235, 0.0926, and 0.0617 g/m^2^ niclosamide). Relative to niclosamide, there were less number of snails that climbed out with tagpo extract.

Lethal concentrations are used to compare activities of different test substances in bioassays. These values are calculated using the Finney method (Probit analysis) or any other appropriate statistical tool [16]. The goodness-of-fit test, also known as the Chi-squared test, is a preliminary statistical treatment that validates the logistic regression model of a dose–response data [17, 18]. If no significant difference is detected between the experimental data and the model, then Probit analysis becomes a valid tool to use in calculating lethal concentrations. The results for the goodness-of-fit test are presented in Table 3. The *χ*^2^ test revealed that all ten dose–response data were fitted to a lognormal distribution with all corresponding *p*-values higher than 0.05.

The calculated lethal concentrations of niclosamide and tagpo extract are presented in Table 4. The LC_50_ value calculated for niclosamide was 0.104 g/m^2^ with *cpt* and 0.127–0.140 g/m^2^ with *mpt*. In terms of LC_90_, niclosamide had a value of 0.266 g/m^2^ using *cpt* and 0.244–0.268 g/m^2^ using *mpt*. For tagpo extract, an LC_50_ value of 1.467 g/m^2^ was calculated using *cpt*. The lowest LC_50_ value calculated with *mpt* was 1.547 g/m^2^ while the highest was 1.797 g/m^2^. The LC_90_ value for tagpo extract was 2.188 g/m^2^ and 2.195–2.501 g/m^2^ determined using *cpt* and *mpt*, respectively.

Lethal ratio test was performed to determine if the difference between paired LC_50_s (or LC_90_s) is due to random errors only. If the confidence limits of a lethal ratio includes 1.000, the difference between lethal concentrations is interpreted as not significant. The lethal ratios and their corresponding confidence limits at 95% confidence level are presented in Table 5. Notably, a significant difference was detected between LC_50_s of *cpt* and *mpt* 1, 0.820 (0.663, 0.977) and LC_50_s of pair *mpt* 1 and *mpt* 3, 0.861 (0.782, 0.939). This means that changing the plate diameter from 85 mm to 21.5 mm has a significant effect on the calculated LC_50_s.

## 4. Discussion

This study reports the performance of new molluscicidal assay based on *O. h. quadrasi* snails using a 12-well plate, 3 snails per well, and a 24 h exposure period. This assay is called miniaturized plate test or *mpt* which is a modification of WHO's conventional plate test (*cpt*). The performance of *mpt* was evaluated using the calculated lethal concentrations of niclosamide and tagpo extract (a plant extract). The method *mpt* uses a smaller amount of test substance and a smaller number of snails compared to *cpt* (Table 1). This implies that the new method may be used to test the molluscicidal activity of substances with very limited sources such as synthetic products. In addition, this new method may be used to increase the throughput of testing crude plant extracts.

Philippines is a biodiversity hotspot, thus bioprospecting for molluscicidal substances from endemic plants and other sources is a worthy endeavor. Novel molluscicides discovered from Philippine natural products active against *O. h. quadrasi* snails such as the tagpo extract can also be tested against other medically important snail species, belonging to the genera *Oncomelania*, *Biomphalaria*, *Pomacea*, *Lymnaea*, and *Bulinus*. For example, tagpo extract is also active against the notorious rice pest, the golden apple snails or *Pomaceacanaliculata* Lamarck, 1819 (unpublished data). Tagpo was discovered through bioprospecting of various Philippine plants for molluscicidal activity.

Similar to *cpt* results, the dose–response data obtained using *mpt* is amenable to rigorous statistical treatment which is a desirable characteristic of a bioassay. Appropriate statistical analyses of dose–response data may be used to detect systematic errors, meaning a suspected bad data due to incompetent snails or contaminated plates may be ruled out by formal *mpt* bioassay with niclosamide. Notably, the calculated LC_90_s for niclosamide using *cpt* and *mpt* are in good agreement with that of Wei et al. [24]. This validates the results obtained in this present study. Another interesting application of *mpt* is in probing of *O. h. quadrasi* snail resistance to widely used molluscicides. For instance, *mpt* can be used to evaluate snail resistance to niclosamide among different geographical isolates of snails in the country, making *mpt* valuable in schistosomiasis research. Taken together, these provide wider applications of this new method.

The significant difference detected in the lethal ratio of paired LC_50_s as seen in Table 5 is a result of snail escaping tendency against niclosamide and the imposed reduction of plate area in *mpt*. This mechanism has been observed for *O. hupensis* snail and other species elsewhere especially at sublethal concentrations of niclosamide [24, 25]. *Oncomelania* snails tend to escape out where niclosamide is present. This behaviour may be thought of a defense mechanism of the snails versus a potentially lethal substance. To add, even without chemical stimulus, snails can escape out of the plate faster in *mpt* than in *cpt* due to a smaller plate area. However, this does not invalidate the results since no significant difference in the LC_90_s was detected with both methods either for niclosamide or tagpo extract (Table 5).

Unlike in WHO's original *cpt* wherein bioassay data are obtained after 4 days, *mpt* generates results after 24 h only [15]. On the one hand, a 4-day exposure period may introduce uncontrolled factors in the test such as contamination of the plate from decomposing snail carcasses, on the other hand, rapid assessment of mortality will ensure that only those fast acting molluscicides will be screened and further developed. In a controlled environment (e.g., *mpt*) the snails are optimally exposed to the active component and an immediate onset of snail-killing effect is expected. Field conditions such as soil absorption capacity, microbial degradation, temperature fluctuation, and rainfall level may dilute the effect of an active compound so that fast-acting molluscicides may be preferred in field application. Temporal variation of molluscicidal activity may be best evaluated in field trials where the average effect of these factors are taken into account.

Moreover, the results also revealed that tagpo extract exhibited potential molluscicidal activity. This is the first report on the molluscicidal activity of an *Ardisia* species. Attempts to further develop this plant extract are currently underway. Our future work would include comprehensive evaluation of the effect of different variables (e.g., exposure period and recovery step) on the accuracy and reproducibility of *mpt*-derived dose–response data.

## 5. Conclusions

This study revealed that the miniaturized plate test, an assay based on *Oncomelania hupensis quadrasi* snails, is an alternative molluscicidal assay to WHO's conventional plate test. This new method offers accurate and reproducible calculation of lethal concentrations. It also uses a small amount of test substance and the dose–response data it generates can be subjected to various statistical tests (i.e., goodness-of-fit test, Probit analysis, and lethal ratio test). Further optimization of *mpt* is a worthy endeavor. Its future use may include the high-throughput testing of natural products (e.g., crude plant extracts) and testing potential resistance of *O. h. quadrasi* snails from different geographical isolates to commonly used molluscicides.

## Figures and Tables

**Table 1:** The amount of substance and number of snails used per trial in conventional (*cpt*) and miniaturized (*mpt*) plate tests.

	Method	Number of test concentrations	Total amount of substance used (g)	Total number of snails used
Niclosamide	*mpt*	7	0.0035	252
	*cpt*	7	0.0171	280

Tagpo extract	*mpt*	5	0.0303	180
	*cpt*	5	0.1459	200

**Table 2:** The snail mortality counts per concentration of niclosamide and tagpo extract using the conventional (*cpt*) and miniaturized (*mpt*) plate tests.

	Concentration (g/m^2^)	*cpt * ^a^	Concentration (g/m^2^)	*mpt* 1^b^	*mpt* 2^b^	*mpt* 3^b^	*mpt* 4^b^
Niclosamide	0.2131	28	0.2330	25	26	26	25
	0.1852	25	0.2000	22	22	22	23
	0.1698	23	0.1833	20	20	20	19
	0.1543	20	0.1667	17	18	17	13
	0.1235	14	0.1333	11	10	10	10
	0.0926	11	0.1000	8	7	6	8
	0.0617	10	0.0667	6	3	3	3
	0.0000^∗^	0^c^	0.0000^∗^	0^d^	0^d^	0^d^	0^d^

Tagpo extract	2.2192	30	2.3963	26	25	25	25
	1.9514	22	2.1072	24	20	19	20
	1.7218	19	1.8593	20	17	17	17
	1.4540	17	1.5700	12	12	8	11
	1.2244	11	1.3221	9	7	1	7
	0.0000^∗^	0^e^	0.0000^∗^	0^f^	0^f^	0^f^	0^f^

^a^Out of 30 snails;

^b^Out of 27 snails;

^c^Out of 70 snails;

^d^Out of 63 snails;

^e^Out of 50 snails;

^f^Out of 45 snails;

^∗^Negative control (water only).

**Table 3:** The results of goodness-of-fit test in terms of Pearson Chi-square statistic for all sets of dose–response data.

	Method	df	*χ* ^2^	*p*-value	Interpretation
Niclosamide	*cpt*	5	6.9962	0.2209	Not significant
	*mpt* 1	5	4.0881	0.5368	Not significant
	*mpt* 2	5	6.9711	0.2228	Not significant
	*mpt* 3	5	2.9620	0.7059	Not significant
	*mpt* 4	5	8.8165	0.1166	Not significant

Tagpo extract	*cpt*	3	6.4780	0.0905	Not significant
	*mpt* 1	3	1.0881	0.7799	Not significant
	*mpt* 2	3	1.5552	0.6696	Not significant
	*mpt* 3	3	2.4043	0.4928	Not significant
	*mpt* 4	3	1.0881	0.7799	Not significant

*cpt* – Conventional plate test; *mpt* – Miniaturized plate test.

**Table 4:** LC_50_ and LC_90_ values in gram per square meter with their 95% confidence limits for niclosamide and tagpo extract against *O. h. quadrasi* snails using conventional (*cpt*) and miniaturized (*mpt*) plate tests.

	Niclosamide		Tagpo extract	
	LC_50_	LC_90_	LC_50_	LC_90_
*cpt*	0.104 (0.088, 0.120)	0.266 (0.191, 0.341)	1.467 (1.356, 1.577)	2.188 (1.892, 2.484)
*mpt* 1	0.127 (0.112, 0.143)	0.268 (0.207, 0.330)	1.547 (1.433, 1.661)	2.195 (1.934, 2.454)
*mpt* 2	0.136 (0.122, 0.149)	0.244 (0.202, 0.285)	1.659 (1.532, 1.786)	2.501 (2.117, 2.885)
*mpt* 3	0.139 (0.125, 0.153)	0.251 (0.207, 0.296)	1.797 (1.700, 1.893)	2.358 (2.127, 2.590)
*mpt* 4	0.140 (0.125, 0.154)	0.261 (0.211, 0.312)	1.659 (1.532, 1.786)	2.501 (2.117, 2.886)

**Table 5:** Lethal dose ratios with their 95% confidence limits for niclosamide and tagpo extract using conventional (*cpt*) and miniaturized (*mpt*) plate tests against *Oncomelania hupensis quadrasi* snails.

Lethal ratio	Niclosamide		Tagpo extract	
	LC_50_	LC_90_	LC_50_	LC_90_
*cpt/mpt* 1	**0.820 (0.663, 0.977)^∗^**	0.991 (0.632, 1.351)	0.948 (0.848, 1.048)	0.997 (0.818, 1.177)
*mpt* 1/*mpt* 2	0.939 (0.793, 1.084)	1.101 (0.787, 1.416)	0.932 (0.833, 1.031)	0.877 (0.707, 1.048)
*mpt* 1/*mpt* 3	0.916 (0.774, 1.057)	1.068 (0.758, 1.377)	**0.861 (0.782, 0.939)^∗^**	0.931 (0.787, 1.074)
*mpt* 1/*mpt* 4	0.912 (0.769, 1.056)	1.026 (0.719, 1.333)	0.932 (0.833, 1.031)	0.877 (0.707, 1.048)
*mpt* 2/*mpt* 3	0.976 (0.841, 1.110)	0.969 (0.729, 1.209)	0.923 (0.837, 1.010)	1.061 (0.867, 1.254)
*mpt* 2/*mpt* 4	0.972 (0.835, 1.109)	0.931 (0.690, 1.172)	1.000 (0.892, 1.108)	1.000 (0.783, 1.217)
*mpt* 3/*mpt* 4	0.996 (0.857, 1.136)	0.961 (0.708, 1.213)	1.083 (0.982, 1.184)	0.943 (0.771, 1.115)

^∗^Difference is interpreted as significant.

## Data Availability

Data available on request. Please contact the corresponding author, Mr. Darwin Gomez. Please email at darwin.gomez@evsu.edu.ph.
